# Glendonite occurrences in the Tremadocian of Baltica: first Early Palaeozoic evidence of massive ikaite precipitation at temperate latitudes

**DOI:** 10.1038/s41598-019-43707-4

**Published:** 2019-05-10

**Authors:** Leonid E. Popov, J. Javier Álvaro, Lars E. Holmer, Heikki Bauert, Mansoureh Ghobadi Pour, Andrei V. Dronov, Oliver Lehnert, Olle Hints, Peep Männik, Zhifei Zhang, Zhiliang Zhang

**Affiliations:** 1 Department of Earth Sciences, National Museum of Wales, Cathays Park, Cardiff, CF10 3NP UK; 2 Instituto de Geociencias (CSIC-UCM), Dr. Severo Ochoa 7, 28040 Madrid, Spain; 3 Department of Earth Sciences, Palaeobiology, SE-752 36 Uppsala, Sweden; 4 Shaanxi Key laboratory of Early Life and Environments, State Key Laboratory of Continental Dynamics and Department of Geology, Northwest University, 710069 Xi’an, China; 5 Department of Geology at Tallinn University of Technology, Ehitajate tee 5, 19086 Tallinn, Estonia; 6 Department of Geology, Faculty of Sciences, Golestan University, Gorgan, 49138-15739 Iran; 7 Geological Institute of Russian Academy of Sciences, 7 Pyzhevskii Lane, Moscow, 119017 Russia; 8 Kazan (Volga Region) Federal University, 18 Kremlevskaya Street, Kazan, 420008 Russia; 9 GeoZentrum Nordbayern, Lithosphere Dynamics, FAU Erlangen-Nürnberg, Schloßgarten 5, D-91054 Erlangen, Germany; 10 Key Laboratory of Economic Stratigraphy and Palaeogeography, Nanjing Institute of Geology and Palaeontology, Chinese Academy of Sciences, 39 East Beijing Road, Nanjing, 210008 China; 11 Faculty of Environmental Sciences, Czech University of Life Sciences Prague, Kamýcká 129, 165 21 Praha 6 Suchdol, Czech Republic; 12 Geological Survey of Estonia, Tartu maantee 85, 10115 Tallinn, Estonia

**Keywords:** Palaeoclimate, Stratigraphy

## Abstract

The Tremadocian (Early Ordovician) is currently considered a time span of greenhouse conditions with tropical water surface temperature estimates, interpolated from oxygen isotopes, approaching 40 °C. In the mid-latitude Baltoscandian Basin, conodonts displaying low δ^18^O values, which suggest high temperatures (>40 °C) in the water column, are in contrast with the discovery of contemporaneous glendonite clusters, a pseudomorph of ikaite (CaCO_3_·6H_2_O) traditionally considered as indicator of near-freezing bottom-water conditions. The massive precipitation of this temperature sensitive mineral is associated with transgressive conditions and high organic productivity. As a result, the lower Tremadocian sediments of Baltoscandia apparently contain both “greenhouse” pelagic signals and near-freezing substrate indicators. This paradox points to other primary controlling mechanisms for ikaite precipitation in kerogenous substrates, such as carbonate alkalinity, pH and Mg/Ca ratios, as recently constrained by laboratory experiments. Preservation of “hot” conodonts embedded in kerogenous shales rich in δ^18^O-depleted glendonites suggests both the onset of sharp thermal stratification patterns in a semi-closed basin and the assumed influence of isotopically depleted freshwater yielded by fluvial systems.

## Introduction

**Figure 1:**
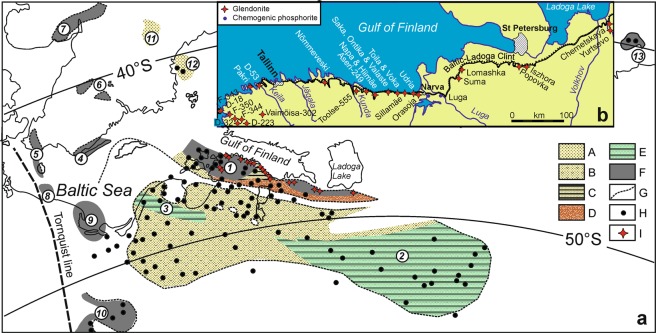
Distribution of major Tremadocian facies in the northern and central parts of East Baltica with setting of glendonite localities; (**a**) facies map (after^5,6^) showing: A, quartzose sand and sandstone (nearshore); B, quartzose sand rich in obolid brachiopods (nearshore); C, fine-grained sand with black shale interbeds (offshore); D, detrital sand with abundant obolid brachiopods (beach and bar systems); E, fine-grained sandstone and shale (offshore to basinal); F, black graptolitic shale (basinal); G, exposure of Tremadocian strata; H, boreholes; I, glendonite localities; geographical areas: 1, North Estonia and St Petersburg region; 2, Moscow Basin; 3, Jelgava depression; 4, Öland and Småland; 5, Scania-Bornholm; 6, Östergötland-Närke-Västergötland; 7, Oslo Region; 8, Łeba area; 9, Łeba-Gdaǹsk area; 10, Podlasie depression; 11, Siljan, 12, Bothnian Sea; 13, Kolguev Island; palaeolatitudes for Tremadocian after^67^; (**b**) schematic map of the Baltic-Ladoga Clint area showing position of glendonite and phosphorite localities; modified after^6^. New (prepared by MGP in Corel Draw 15, https://www.corel.com/en/products/coreldraw/).

**Figure 2:**
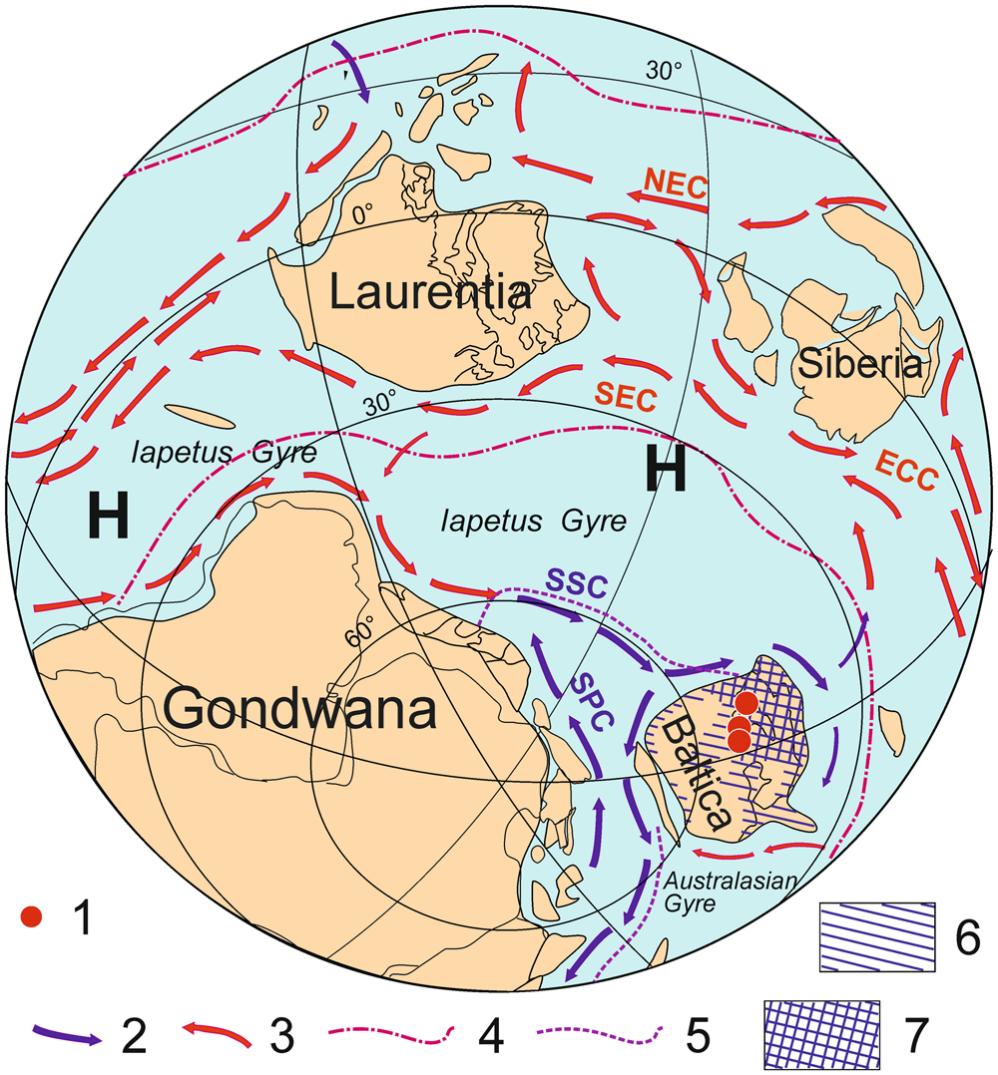
Early Tremadocian schematic palaeogeographical reconstruction for the South Hemisphere, including inferred oceanic circulation (modified data on patterns of circulation after^68^). Legend: 1, areas of ikaite precipitation; 2, cold water currents; 3, warm water currents; 4; Tropical Convergence; 5, Polar Convergence; 6, inferred areas of epeiric seas on Baltica continent; 7, Baltoscandian Basin; NEC, North Equatorial Current; SEC, South Equatorial Current; ECC, Equatorial Counter Current; SSC - South Subpolar Current; SPC - South Polar Current; H, subtropical high pressure zones; K, Kolguyev Island; T, Timan – Pechory Region; CA, Central Africa. New; (prepared by MGP in Corel Draw 15, https://www.corel.com/en/products/coreldraw/)

Except one rather controversial note^1^, the record of glendonites displays an apparent gap from Neopoterozoic^2^ to Permian^3^ times. However, similar calcareous nodular aggregates embedded in Tremadocian black shales of the East Baltic (Fig. 1a), the so-called “antraconites”, have been known for more than 150 years. These aggregates are documented from 24 geographical localities in the Türisalu and Koporiye formations (*Cordylodus angulatus* - *Paltodus deltifer pristinus* zones) and sporadically in the Orasoja Member (upper part of the Kallavere Formation; *Cordylodus angulatus* - *Paltodus deltifer pristinus* zones), exposed along 600 km of the Baltic-Ladoga Glint^4^, a transect linking North Estonia to the eastern St Petersburg area (Fig. 1b). All these units were accumulated in the Baltoscandian Basin (Fig. 2), an epeiric sea with a central flat-floored depocentre rimmed to the south (recent coordinates) by a chain of low islands and associated shoal complexes^5,6^. During Tremadocian times, the basinal depocentre^4,7^ recorded black shale deposition episodically punctuated by wave and storm-induced processes, pointing to a sediment-starved, offshore-to-basinal clayey substrate, in which organic matter and trace metals became highly concentrated due to extremely low deposition rates and an exceptionally low influx of siliciclastic material^8^. In contrast, nearshore environments comprised uncemented, well-washed, cross-laminated quartzose sands, which included high concentrations of allochthonous obolid coquinas (e.g. Rakvere phosphorite ore deposit) that were continuously reworked along the shorelines. Major Furongian–Tremadocian regressions are recognized in these nearshore environments by the presence of distinct stratigraphic gaps (Supplement. Fig. 1), some of them highlighted by the evidence of palaeokarst^9^.

## Sedimentary and palaeoecological background of glendonite-bearing strata

The above-reported inner-platform phosphoritic (wt. P_2_O_5_ >18%) bars contain *Rhabdinopora* graptolites^5^, which leave no doubts that they were synchronously deposited with the black shales of the glendonite-bearing Türisalu Formation. These phosphatic bars played a significant source for the increasing phosphate pollution of the Baltoscandian semi-closed Basin, especially when high alkaline dysaerobic conditions developed at the sediment-water interface^5,10,11^. Episodic water stratification, controlled by temperature and density, has been proposed for the Baltoscandian Basin to explain modifications in TOC concentration and accumulation of metalliferous ore deposits^12,13^.

The kerogenous and metalliferous black shales of the Türisalu and Koporiye formations display shallowing-upward parasequences, up to 7 m thick. Massive to laminated black shales are topped by siltstone interbeds rich in cross- and wavy-lamination, symmetric ripple marks, centimetre-scale lag deposits with grading, scouring surfaces and episodic record of burrowing and spiculites^14–16^. The parasequences are associated with vertical decimetre-scale redox-sensitive trace metal shifts^17–19^ reflecting metal sequestration related to contemporaneous fluctuations in sedimentation rate. In NE Estonia, the Toolse Member (Türisalu Formation) becomes thinner and consists of a centimetre-thick alternation of siltstone and black shale containing authigenic carbonate and sulphide mineralizations^20,21^. In Scania, the influence of relative high-order sea-level fluctuations is recognized by the vertical stacking pattern of different claystone-dominant facies associations of the Alum Shale^22^. In the parasequences of the Türisalu and Koporiye formations, glendonites are relatively common in the lower massive-to-laminated black shales and in the black shale interbeds of the upper part. The latter contain scattered centimetre-thick lag deposits rich in clasts derived from glendonites pointing to the influence of storm-induced episodes.

Oxygen content was also variable, ranging from temporary oxygenated conditions above the sediment-water interface (supported by the episodic occurrence of ichnofossils, often preserved as a result of pyrite infill, and thin, lens-like layers of spiculites and associated acrotretid brachiopods^5,16^) to dysaerobic, high alkaline conditions proved by the enrichment in redox-sensitive metals^8^, including molybdenum, uranium and vanadium. Oxygenation episodes are recognized by the sudden development of metazoan colonization, burrowing and development of microbially induced sedimentary structures^16,23^. Microbial mats were then able to stabilise the seafloor, protecting against erosion and increasing the cohesiveness of the marine substrate. It is noticeable the local abundance of *Kinneya*-type wrinkle structures, considered as subsurface structures developed on a clayey substrate underneath biofilms and mats^24,25^.

Since Miaolingian to Tremadocian times, the coastal plains  and shoals of the Baltoscandian Basin were inhabited by low diversity benthic communities dominated by a single or few linguliform brachiopod species of the genera *Obolus*, *Oepikites*, *Schmidtites* and *Ungula*, characterized by organophosphatic shell mineralization^4,5^, commonly associated with *Skolithos* trace fossils. These obolid-dominant communities adapted to soft, mobile substrates affected by storms and tidal currents. Their gradual proliferation reached its peak during the latest Furongian (*Cordylodus andresi* and *Cordylodus proavus* zones), when vast accumulations of phosphatic shelly material deposited nearshore. Subsequently, these brachiopod communities disappeared during the early Tremadocian and were replaced by hexactinellid sponges and the micromorphic linguliform brachiopod *Eurytreta*, which are considered disaster taxa postdating an extinction event that affected the late Furongian shelly communities^4^. The extinction event closely coincided with major marine transgression (*Cordylodus angulatus* Zone) that led to continuous deposition of black shales. The early planktonic graptolites *Rhabdinopora* became a common component of the pelagic fauna in the early Tremadocian Baltoscandian Basin, while a low diversity conodont fauna, dominated by *Cordylodus* spp., were not significantly affected by environmental changes^4,5^.

The Furongian–Tremadocian Nd isotopic signatures show median values of the *ε*Nd(t) within the range from −7.0 to −8.0 for the whole Baltoscandian Basin^26^. These data were probably close to the original signatures of the adjacent Iapetus oceanic water masses, suggesting free exchange: there are no signs of the high negative *ε*Nd(t) valued characteristic of old cratons. Therefore, a significant part of the Baltic continent, including the Fennoscandian Shield, was covered by epeiric seas. Sedimentation rates of the East Baltic black shales were extremely low, below 10 mm per millennium and approaching those of pelagic clays in present-day oceans^16,27^.

## Updated significance of Tremadocian antraconites

**Figure 3:**
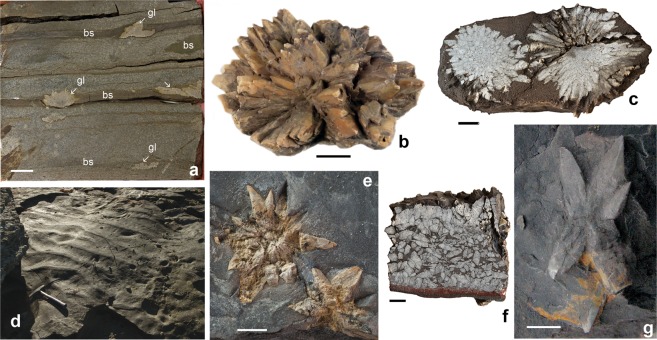
Photographs of glendonites and sedimentary features from the East Baltic black shales; (**a**) GIT 366-296, section of a drill core showing silty sandstone alternating with thin layers of black shale (bs) bearing glendonites (gl), D-223 borehole; (**b**) rosette cluster of glendonite, Toolse Member (*Paltodus deltifer pristinus* Zone), Napa, Estonia; (**c**,**f**,**h**) cross sectional view of two rosette glendonite clusters, cross sectional view of “glendonite bed”, and stellate glendonite cluster, Koporiye Formation (*Cordylodus angulatus* Zone), Syas River near Yurtzevo, Russia; (**d**) bedding surface of black shale with wave generated symmetrical ripple marks, Tabasalu Member (*Cordylodus angulatus* Zone), Pakri Cape; (**e**) GIT 571-19, two stellate glendonite clusters, Tabasalu Member (*Cordylodus angulatus* Zone), D-32 borehore, Vidruka; (**g**) TUG 220-77b, blade-shaped glendonite, Tabasalu Member, F-350 borehole, Kirimäe; (**b**,**d**–**f**) scale bars are 1 cm; (**e**,**g**) scale bars are 5 mm; (**a–g**) photo by H. Bauert; (**h**) photo by A. Dronov.

Antraconitic aggregates were sampled in the Tremadocian Türisalu Formation of North Estonia and the Koporiye Formation of the eastern St Petersburg region (Fig. 1b; Supplement. Figs 2–3). Antraconites occur as single crystal pseudomorphs, stellate (Fig. 3e,g) and rosette (Fig. 3b,c) clusters, usually 5–10 cm across and up to 20 cm in length, encased in black shales. One characteristic glendonite horizon, 15 cm thick, embedded in a distinct grey graptolitic clay of the Koporiye Formation, is traceable over 3.5 km on the eastern bank of the Syas River, between Chernetshkaya and Yurtzevo villages^5^ (Fig. 3b). It represents a compact aggregate of crystals, with randomly oriented long axes marking the top of a pyritised sandstone layer (Fig. 3f). The precipitation of ikaite probably occurred at the sediment-water interface, highlighting the top of a condensed bed.

**Figure 4:**
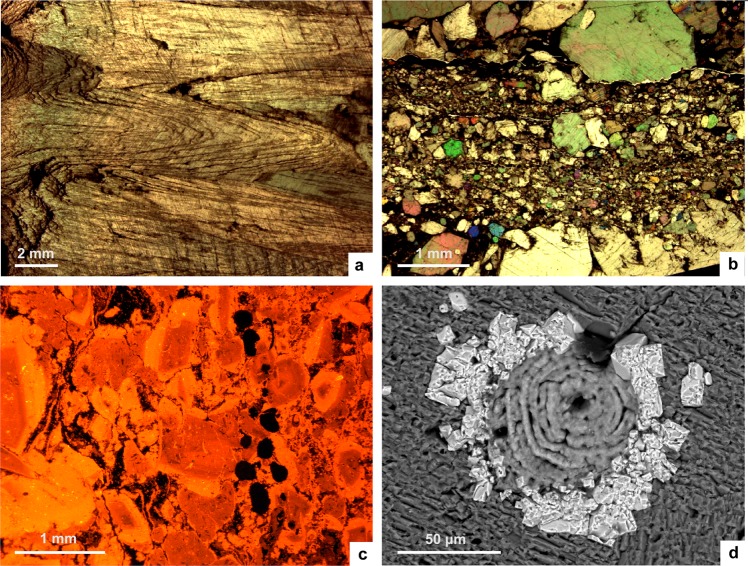
Thin-section photomicrographs of glendonite and associated sediment; (**a**) core of glendonite displaying the prismatic chevron-like habit of stellate pseudomorphs, Koporye Formation of St Petersburg region (petrographic microscope, plane light); (**b**) glendonite showing mosaics of microgranular calcite after recrystallization of original ikaite (petrographic microscope, parallel light), Türisalu Formation, North Estonia; (**c**) cathodoluminescence image of previous picture showing zoned ovoidal calcite grains with red-CL colours surrounded by subsequent orange-CL overgrowths; (**d**) SEM image showing euhedral crystals of pyrite nucleated on external wall of a phosphatic linguliform brachiopod, Koporiye Formation of St Petersburg region.

Glendonite crystals display a slightly distorted, prismatic habit (Fig. 4a), grading both laterally and centripetally into microgranular mosaics of calcite (Fig. 4b), commonly red-stained by dispersed ferroan oxi-hydroxides and variable content in organic matter. XRD and geochemical analyses reveal a predominance of calcite and low-Mg calcite (Mg/Ca ratio < 1.2^28,29^), locally contaminated by silty quartz and feldspar and the scattered presence of dolomite rhombs. Cathodoluminescence (CL) petrography distinguishes rhombohedral, fine-fibrous, chevron-like, ovoidal, spherulitic and clear mosaics of calcite (Fig. 4c). Crosscutting relationships between different calcite phases suggest multiple stages of dissolution-recrystallization and (either complete or partial) replacement of pre-existing types of calcite. Successive recrystallization phases from an ikaite precursor are marked by non-luminescent to dull brown-orange (bladed to equant) and reddish (granular mosaics) for younger neomorphic calcite mosaics. CL patterns reflect both a progressive increase and zonation in luminescence of the youngest calcite generations. This progressive increase in luminescence activators may be related to changes from relatively oxidizing (or suboxic) ground water to more reducing pore waters.

The wrapping of shale laminae around the glendonite crystals demonstrates that the concretions and nodules lithified during early diagenesis. However, competency differences caused by variable early-lithification of crystal aggregates and interbedded claystone contributed to fracturing. Two kinds of fissures can be distinguished. Those unrecognizable under CL are up to 2 mm wide, have no preferential alignment and are commonly branched and anastomosing; walls are highly irregular and show a very poor (or absent) fit. These fractures formed in a soft, semi-cohesive substrate (Fig. 3a,c). In contrast, fissures easily detectable under CL, up to 1 cm wide, have their walls dominantly composed of straight segments that exhibit a clear fit. Their porosity was occluded by a CL bright yellowish cement. They formed at a late stage of diagenesis in fully lithified concretions.

The dominant form of dolomitization occurs as euhedral to subeuhedral, dolomite rhombs, up to 60 μm in size. Locally, pervasive dolomitization has led to equigranular mosaics with little trace of primary textures. Fine-grained pyrite, identifiable as cubic, anhedral and framboidal forms and up to 8 μm in size, are locally abundant throughout the concretions and the encasing matrix. Occasionally, pyrite directly encrusted previous hard substrates, such as scattered fossil skeletons (Fig. 4d).

## Geochemical data of major elements from glendonites

After the ikaite → glendonite transformation, the precipitation and subsequent recrystallization of the calcite pseudomorph can be illustrated and characterized geochemically. Primary glendonites have typically almost Mg-free calcite as first crystallization phase, and then as later filling phases alternating low-Mg calcite and calcite phases. Compositional zoning in calcite to low-Mg calcite crystals is geochemically characterized by differences in the MgO/CaO ratio ranging from 0.008 to 0.55, respectively. Chemical modifications are distinct: low-Mg calcite is considerable enriched over calcite in Mn and Sr content (×2) and Fe (×10), between crystallographically nonequivalent, time-synchronous growth sectors (Supplement. Table 1). The heterogeneity in Mg, Fe, Mn and Sr composition can be illustrated by the above-reported distinct increases in luminescence activators (CL), and by BSE-SEM images showing zoned calcite to low-Mg calcite growth sectors of primary glendonite (Supplement. Fig. 4A,B).

## Isotope data from glendonites

**Figure 5:**
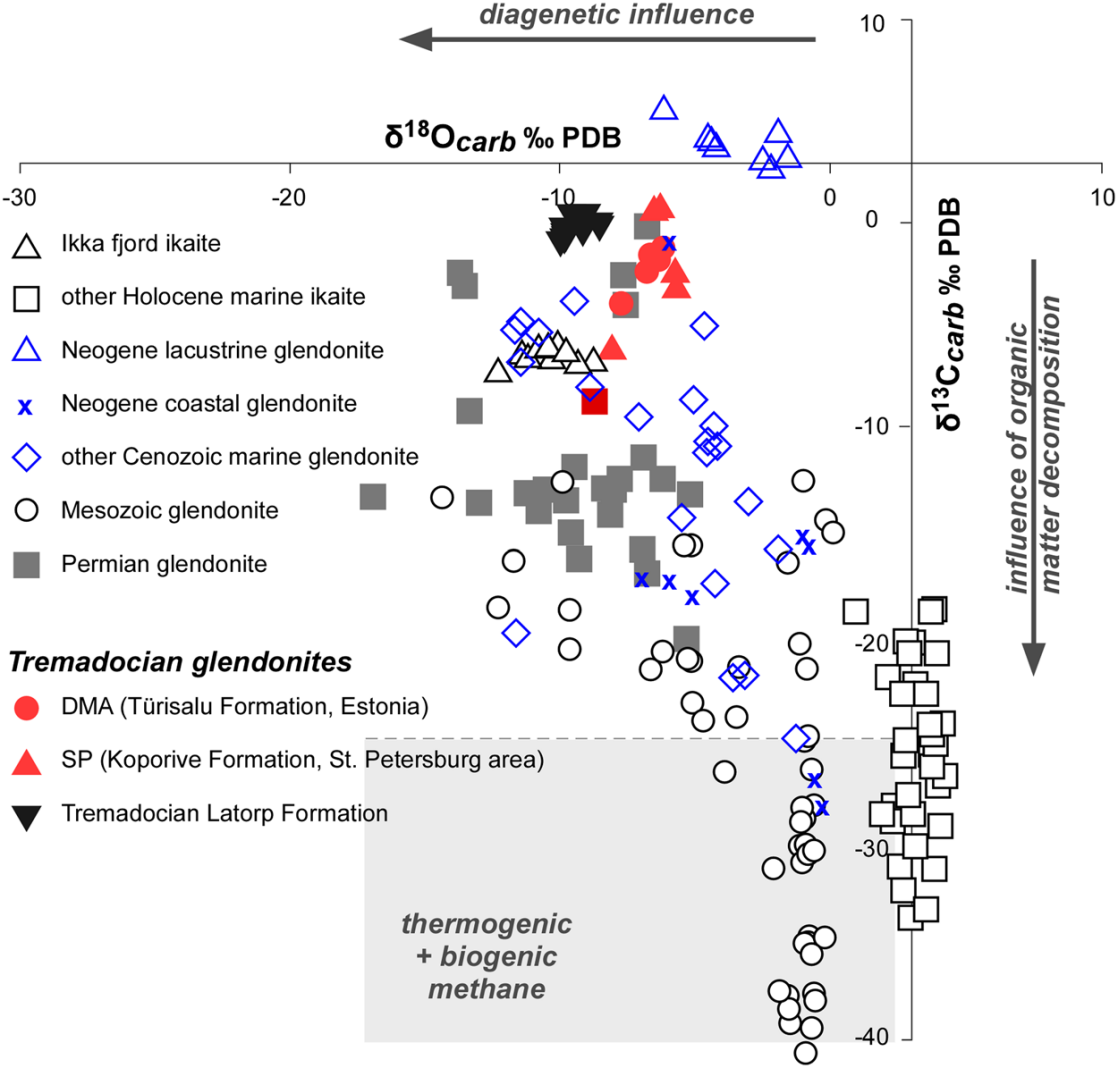
Stable isotope dataset plot of glendonite and ikaite. Ordovician data in Repository Data; chemostratigraphy of the Latorp Formation after^36^; remaining data after^3,31,35,39,69^.

Carbon and oxygen isotope analyses were carried out both in the bladed and microgranular crystals of glendonitic calcite and low-Mg calcite in order to characterize the earliest diagenetic phases. The results are presented in Supplement. Table 2 and graphically shown on Fig. 5. Isotopic data cluster in a single field, where carbon isotope ratios range from +0.6 to −8.9‰ (the bladed crystals being the most enriched in ^18^O and depleted in ^13^C) and similar oxygen values from −5.7 to −8.2‰. These data are consistent with isotope data derived from other methane-free glendonites^3,30–32^. Permian glendonites described in Australia^33^ display greatest isotopic heterogeneities (ranging up to 25‰ in ^13^C and ^18^O) as a result of complex early-diagenetic overprints; their bladed-to-equant microgranular calcite, interpreted as the primary ikaite replacement, also displays the most enriched ^18^O and depleted ^13^C values of the successive calcite cements.

Recent glendonites typically show a much broader range in carbon isotope values, ranging from +10 to −40‰^3,31–35^ (Fig. 5), which are strongly dependent on the depositional environment: the extremely negative δ^13^C values (<−20‰) of many deep marine glendonites are likely controlled by the input of biogenic methane, whereas ikaite precipitated in lacustrine environments exhibits positive δ^13^C values.

Late Tremadocian to Floian glendonite-free limestone interbeds sampled in the Latorp Formation (Jämtland, Sweden), a laterally equivalent of the overlying glendonite-free glauconitic sands of the Leetse Formation, have yielded δ^13^C values heavier than those of the study glendonites (ranging from +0.27 to −0.73‰) but lighter δ^18^O signatures (from −8.29 to −9.89‰)^36^, reflecting a stronger diagenetic overprint of the succession in the glendonitic-free depocentre of the Baltoscandian Basin.

The oxygen isotopes derived from the Baltic glendonites are not used here for palaeotemperature purposes. As the ikaite → calcite transition results in a 68.6% volume loss, related to the release of structural water from the original ikaite crystal^34^, the resulting glendonite is controlled by pseudomorphic transformation into calcite and subsequent porosity occlusion by sparry mosaics of calcite and low-Mg calcite, with different oxygen and carbon isotopic compositions than the original ikaite^30^. Palaeotemperature interpolations of glendonite probably reflect the ikaite decomposition temperature, not the ikaite precipitation temperature^33,37^.

## Isotope data from contemporaneous conodonts

Biogenic phosphate of three conodont samples from the glendonite-bearing Orasoja and Toolse members of the Toolse 555 drill core, in the vicinity of Kunda town, were analysed for oxygen isotopes (Fig. 1b; Supplement. Fig. 2 and Table 3). Conodont elements are scattered on the bedding surfaces of exposed black shales, but they were not used in the analysis because of strong impregnation with iron oxides. δ^18^O values obtained from the corresponding levels A2 (+14.4‰; *C. angulatus* Zone), B3–4 (+14.4‰, *C. angulatus* Zone) and C (+14.7‰; *P. deltifer pristinus* Zone) from this core^38^ were homogeneously low.

The absolute temperatures derived from the δ^18^O values vary according to different equations: the two δ^18^O values (both 14.4‰) obtained from the *C. angulatus* Zone calibrated to NBC 120c (V-SMOV) = 21.7‰ translate 49.9 °C^39^ and 48.1 °C^40^ (V-SMOV = −1). The value based on selected *Paltodus* material from the lower *P. deltifer* Zone (*P. d. pristinus* Subzone), calculated with the same equations, translates to 48.6 °C and 46.8 °C, respectively.

### Constraining ikaite vs. δ^18^O_[phosphate]_ for reconstructing Tremadocian palaeoceanographic conditions

Glendonites are not informative of sedimentary environments. They have been reported from lacustrine and littoral to bathyal (~4000 m) environments, springs, melted sea ice and even caves^35^. In the Tremadocian of the Baltoscandian Basin, the preservation of centimetre-thick lag intervals, rich in glendonite-derived clasts, associated with symmetrical ripples marks and microbially induced sedimentary structures (Supplement. Fig. 5), points to the episodic influence of storm-induced processes reworking the calm-water substrates that served for ikaite nucleation. Therefore, some Baltic glendonites nucleated at bathymetries shallower than the maximum depth of the storm wave base.

Ikaite precipitation is favoured by environments characterized by low temperature (below 4 °C), high pH, high alkalinity, elevated concentration of phosphate and organic-rich marine substrates, where methane oxidation can take place. As a result, the occurrence of glendonite rosette clusters in calm-water clayey substrates has been traditionally considered as one of the most reliable palaeotemperature indicators of near-freezing conditions^3,41^. However, recent experiments^42–44^ have demonstrated that the main chemical factors that control ikaite precipitation are pH and salinity, as a result of which ikaite can precipitate at temperatures up to 12 °C. These results question the suitability of glendonitic calcite pseudomorphs as a proxy for near-freezing conditions. Ikaite unrelated to near-freezing conditions has been discovered in Recent kerogenous sediments where high carbonate alkalinity concentrations and elevated pH conditions (up to 10) are directly controlled by the anaerobic oxidation of organic matter and the photosynthetic activity of microbial communities^43,45,46^.

In our case study, the massive precipitation of ikaite is not contemporaneous with the presence of glaciomarine deposits close to the South Pole^47^. It is also in obvious discrepancy with the palaeotemperature interpolations suggested by the stable oxygen isotope signatures obtained from contemporaneous biogenic calcite (brachiopods) and apatite (linguliform brachiopods and conodonts) sampled in subtropical substrates suggesting higher temperatures (up to 40 °C) for both the seafloor and the water column^48,49^, as well as by the above-reported conodonts from the Toolse 555 drill core.

Isotope data from conodont apatite of subtropical Laurentia^50^ show high δ^18^O values across the Furongian–Tremadocian transition, depleted values (reflecting some strong warming) into the uppermost *angulatus*/lowermost *manitouensis* Zone (coeval with the basal *deltifer* Zone in Baltica), followed by a positive shift starting in the lower *manitouensis* Zone and continuing into and throughout the Floian with minor fluctuations through the late Floian. This rise in δ^18^O values reflects some long-term cooling starting at a level comparable to the lowermost *deltifer* Zone in Baltoscandia^51^. The scale of the relative negative-positive shift of more than 2‰ in δ^18^O during the lower Tremadocian *fluctivagus*/*angulatus* interval is comparable with glaciation vs. deglaciation turnovers recorded during the past 110,000 years^52^.

Isotope data from the East Baltic require some explanation. Unusually low δ^18^O values from biogenic (conodont) phosphates of the glendonite-bearing Orasoja and Toolse members can be explained by either significant freshwater influx from surrounding land or high surface water temperatures pointing to sharp density stratification patterns of the water column. The significant differences in δ^18^O values obtained for the water column and the water/sediment interface in fossil record are rare but not exceptional. A close analogy can be found in unusually light δ^18^O values, obtained from some shark teeth across the Palaeocene–Eocene transition in the area of North Sea^53^. In both cases the observed paradox can be explained by significant decrease in surface water salinity caused by increased fresh water fluvial discharge and sharp stratification of the water column. A high-latitude present-day analogue is the freshwater influx of Siberian rivers, bearing extremely low δ^18^O values into the Arctic shelves^54,55^. Due to the mid-latitude position of Baltica in the Tremadocian, low δ^18^O values of freshwater runoff into the semi-closed Baltoscandian epeiric sea should be expected, because the oxygen isotopy yielded by rivers is drastically controlled by latitude and physical oceanography^56,57^. Yet, strong deficit of siliciclastic supply in the basin together with the above-reported Nd isotopic signatures^26^ show no signs of the high negative εNd(t) values characteristic of craton sources. Further evidence is yielded by the conspicuous occurrence of the free floating planktonic green algae *Botryococcus*, which occur in Sweden through the Alum Shale Formation, including its uppermost Tremadocian part. These oil-producer algae are considered as indicators of at least freshwater influence, due to its abundance in both brackish and lacustrine environments^58^.

Palaeotemperature interpolations derived from oxygen isotopes in bioapatite should take into account possible secular changes in the oxygen isotope composition of Phanerozoic seawater^59^, which could be responsible for apparently too high scores of inferred sea-water temperatures, based on different present-date-based formulas. Furthermore, recent developments in biomineralisation studies clearly demonstrate that biogenic hydroxyapatite is metastable outside the animal body^60,61^, which creates new challenges in application of biogenic phosphates for interpretation of isotope analysis outcomes and requires better understanding of their chemical taphonomy.

Finally, long-term episodes of thermally stratified water masses have been proposed for the Lower Ordovician Baltoscandian Basin to explain the widespread development of kerogenous shales rich trace-metal ore deposits and distinct separation of pelagic fossil taxa^12,13^. Among them conodonts, vertebrates with high rate metabolism that adapted to well-oxygenated surface waters, whereas graptolites exhibited signs of adaptation to feed on nutrient-rich bacterioplankton based food chain along the boundary between oxygen-rich surface waters and anoxic waters at depth below the thermocline^62^.

## Concluding remarks

The massive precipitation of ikaite in the Tremadocian black shales of North Estonia and the adjacent St Petersburg region of Russia, which lasted ca. 5 m.y., is exceptional and the first ever recorded for the entire Cambrian–Carboniferous sedimentary record worldwide. It took place in a semi-closed, epeiric sea located at temperate southern latitudes, and was associated with transgressive conditions and high organic productivity.

The isotopic data of the reported glendonites represent a mixture of pseudomorphic transformation into calcite and occlusion of the porosity related to the ikaite → glendonite transformation by early-diagenetic calcite and low-Mg calcite, the latter displaying higher contents in Mn, Fe and Sr. In contrast, the low δ^18^O_apatite_ values derived from conodonts living in the water column and fossilised in glendonite-bearing strata, interpolate to high temperatures (>40 °C), comparable to those reported in contemporaneous calcitic brachiopods from shallow-water subtropical settings.

The preservation of “hot” conodonts in glendonite-bearing shales points to other primary controlling mechanisms for ikaite precipitation in kerogenous substrates, such as carbonate alkalinity, pH and Mg/Ca ratios in the pore-water, as recently confirmed by laboratory experiments. The resulting gradient in palaeotemperature of the Tremadocian water column may have been biased by the onset of thermal stratification in the semi-closed Baltoscandian Basin and probable freshwater influx, whereas secular changes in the oxygen isotope composition of Phanerozoic seawater should not be discarded. The thermal/saline stratification of the Baltoscandian water column should have played an important role in moderating subpolar climates and reducing latitudinal gradients in Tremadocian times.

## Methods

The analysed antraconites were sampled in the Türisalu Formation (*Paltodus deltifer pristinus* Zone) of a costal section in vicinity of Udria, North Estonia (acronym DMA), the Koporiye Formation (*Cordylodus angulatus* Zone) close to Yurtsevo village, and the St Petersburg region (SP). The greatest concentration of antraconites occurs within a bed, 1 m thick, at Yurtsevo and Chernetskaya sections (Fig. 1b, Supplement. Fig. 2; see also GPS settings in Repository Data).

Samples were petrographically characterized using a combination of methods, including transmitted light microscopy with thin-sections stained by Alizarin Red S and Potassium Ferricyanide, scanning electron microscopy (SEM at Museo Nacional de Ciencias Naturales, Madrid) operating in back-scattered electron (BSE) image and energy dispersive X-ray (EDS) analysis, and separate cold cathodoluminescence microscopy (CL at Instituto Geológico y Minero de España, Tres Cantos). Analytical results of back-scattered electron imaging and EDS analyses display an error of ±5 to 7%. The qualitative mineralogical composition of some complex samples was determined by the X-ray powder diffraction method. Interpretation of cementation history was made by distinguishing cement types based on colour, brightness, luminescence patterns, cement morphology and cross-cut relationships. Complex zonation of cements (revealed by CL, BSE and EDS) allowed correlation of cement zones between samples. Major elements were determined using X-ray fluorescence and inductively coupled plasma mass spectrometry (ICP-MS at AcmeLabs, Canada). Precision for major elements is usually better than 2%, 5–10% and 3–7%, respectively.

Stable isotopes of oxygen and carbon for whole-rock carbonates were removed by dental drill under a binocular microscope and analysed at Erlangen University. Carbonate powders were reacted with 100% phosphoric acid at 75 °C using a Kiel III carbonate preparation line connected online to a ThermoFinnigan 252 mass spectrometer. All values are reported in permil relative to V-PDB by assigning a δ^13^C value of +1.95‰ and a δ^18^O value of −2.20‰ to NBS19. Reproducibility was checked by replicate analyses of laboratory standards and is better than ±0.06‰ (1 std.dev.).

Chemical conversion of the phosphate bound in conodont apatite into trisilverphosphate (Ag_3_PO_4_) was performed following^63^ method and subsequent oxygen isotope analyses were performed using a TC‐EA (high-temperature conversion-elemental analyzer) coupled online to a ThermoFinnigan Delta V Plus mass spectrometer. 0.2 to 0.3 mg Ag_3_PO_4_ was weighed into silver foil and transferred to the sample carousel of the TC‐EA. At 1450 °C, the silver phosphate is reduced and CO forms as the analyte gas^64^. CO was transferred in a helium stream through a gas chromatograph via a Conflo III interface to the mass spectrometer and values are reported in ‰ relative to VSMOW. Samples as well as standards were measured in triplicate, measurements were calibrated by performing a two‐point calibration^65^ using NBS 120c (21.7‰) and a commercial Ag_3_PO_4_ (9.9‰). A laboratory standard was used as a control standard and processed together with the samples. All standards were calibrated to TU1 (21.11‰) and TU2 (5.45‰^66^). External reproducibility, monitored by replicate analyses of samples was ±0.14 to ±0.29‰ (1 σ).

## Supplementary information

Glendonite occurrences in the Tremadocian of Baltica - supplement
