# Supplementary material for: Glendonite occurrences in the Tremadocian of Baltica: first Early Palaeozoic evidence of massive ikaite precipitation at temperate latitudes
Source: Sci Rep. 2019 May 10;9:7205. doi: 10.1038/s41598-019-43707-4 (PMC6510892; doi:10.1038/s41598-019-43707-4)
Supplement: Supplementary file 1 — Glendonite occurrences in the Tremadocian of Baltica - supplement [file 41598_2019_43707_MOESM1_ESM.pdf]

Glendonite occurrences in the Tremadocian of Baltica: first Early Palaeozoic evidence of massive ikaite precipitation in temperate latitudes

Leonid E. Popov[1], J. Javier Álvaro[2], Lars E. Holmer[3,4], Heikki Bauert[5], Mansoureh Ghobadi Pour[1,3,6], Andrei V. Dronov[7,8], Oliver Lehnert[9-11], Olle Hints[5], Peep Männik[5], Zhifei Zhang[4], Zhiliang Zhang[4]

[1] Department of Earth Sciences, National Museum of Wales, Cathays Park, Cardiff CF10 3NP, UK

[2] Instituto de Geociencias (CSIC-UCM), Dr. Severo Ochoa 7, 28040 Madrid, Spain, jj.alvaro@csic.es

[3] Department of Earth Sciences, Palaeobiology, SE-752 36 Uppsala, Sweden, lars.holmer@pal.uu.se

[4] Shaanxi Key laboratory of Early Life and Environments, State Key Laboratory of Continental Dynamics and Department of Geology, Northwest University, 710069, Xi'an, China

[5] Institute of Geology at Tallinn University of Technology, Ehitajate tee 5, 19086 Tallinn, Estonia, heikki.bauert@ttu.ee, olle.hints@ttu.ee, *peep.mannik*@ttu.ee

[6] Department of Geology, Faculty of Sciences, Golestan University, Gorgan 49138-15739, Iran

[7] Geological Institute of Russian Academy of Sciences, 7 Pyzhevskii Lane, Moscow 119017, Russia, dronov@ginras.ru

[8] Kazan (Volga Region) Federal University, 18 Kremlevskaya Street, Kazan 420008, Russia

[9] GeoZentrum Nordbayern, Lithosphere Dynamics, FAU Erlangen-Nürnberg, Schloßgarten 5, D-91054 Erlangen, Germany, oliverlehnert@yahoo.de

[10] Key Laboratory of Economic Stratigraphy and Palaeogeography, Nanjing Institute of Geology and Palaeontology, Chinese Academy of Sciences, 39 East Beijing Road, Nanjing 210008, China

[11] Faculty of Environmental Sciences, Czech University of Life Sciences Prague, Kamýcká 129, 165 21 Praha 6 – Suchdol, Czech Republic

*Correspondence to lars.holmer@pal.uu.se

SUPPLEMENTARY DATA

Supplement. Fig. 1. **Revised lithostratigraphical correlation chart for the Cambrian (Miaolingian) - Ordovician (Tremadocian) of North Estonia and European Russia; modified from [5].** New (prepared by MGP in Corel Draw 15, https://www.corel.com/en/products/coreldraw/).

Supplement. Fig. 2. Lower Tremadocian stratigraphical logs sampled for glendonites and conodont biogenic phosphates; (a) drill core of T-555 borehole in vicinity of Kunda (Fig. 1b) [38], A, B, C show stratigraphical intervals of conodont samples used in the oxygen isotope analysis; **(b)** Koporiye Formation in vicinity of Kolchanovo and Yurtzevo, showing glendonite occurrences; **(c)** Orasoja Member of Kallavere Formation and Toolse Member of Türisalu Formation showing setting of glendonite samples; modified from [5]. New (prepared by MGP in Corel Draw 15, https://www.corel.com/en/products/coreldraw/).

Supplement. Fig. 3. Geological cross section through Furongian-Tremadocian deposits in North Estonia. Legend: 1, glauconitic sands of Leetse Formation (Floian); 2, glauconitic clays of Varangu Formation (Tremadocian); 3, black shales of Türisalu Formation (Tremadocian); 4-12, Kallavere Formation (upper Furongian-Tremadocian); 4, Orasoja Member of black shale interbeds (P_2O_5<3%); 5, Vikhula Member of fine grained sands with reworked obolid shells and subsidiary black shales (P_2O_5=1-3%); 6, 7, Rannu Member of quartzose sands (P_2O_5 =3-9%) and detrital phosphoritic sands (P_2O_5>9%); 8, Suurjõgi Member of quartzose sands often with bidirectional cross-lamination (P_2O_5=3-9%); 9, 10, Maardu Member of silty sands with subsidiary black shales (P_2O_5=1-3%) and coquina accumulations (P_2O_5>18%); 11, Ülgase Formation (lower Furongian) of silts and silty clay; 12, Tiskre Formation (Cambrian Series 2). Zero datum level coincides with the inferred position of the Cambrian–Ordovician boundary[5] (modified). New (prepared by MGP in Corel Draw 15, https://www.corel.com/en/products/coreldraw/).

Supplement. Fig. 4. BSE-SEM images of the calcite (ca) to low-Mg calcite (LMC) zoned growths in the pseudomporh glendonite; py - pyrite.

Supplement. Fig. 5. View of upper-bedding surfaces resembling *Kinneya* wrinkle structures showing meandering and partly interfering, flat-topped to rounded crests and intervening grooves and pits; **(a)** with higher and **(b)** lower density patterns; base of Türisalu Formation at Pakri cape, Estonia; scale arrowed.

Supplement. Table 1. Major element composition of glendonite calcite.
Supplement. Table 2. Carbon and Oxygen isotope data from the glendonites from the Türisalu Formnation (Udria, DMA) and the surroundings of St Petersburg (SP).
Supplement. Table 3. $\delta^{18}O$ data from conodont apatite (NSB 120c = 21.7 ‰) sampled in the Toolse 555 drill core".

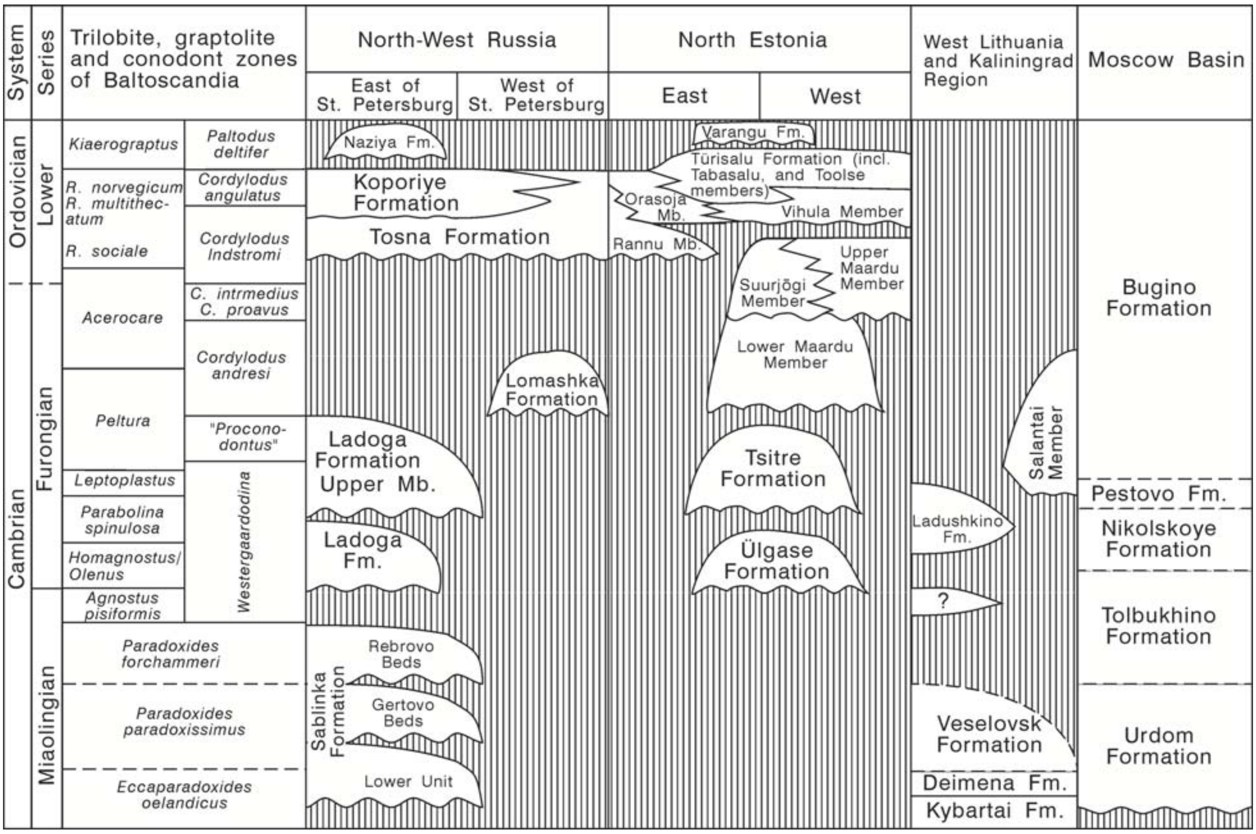
System
Series
Trilobite, graptolite and conodont zones of Baltoscandia
North-West Russia
East of St. Petersburg
West of St. Petersburg
North Estonia
East
West
West Lithuania and Kaliningrad Region
Moscow Basin
Ordovician
Lower
Cambrian
Furongian
Miaolingian
Kiaerograptus
Paltodus deltifer
R. norvegicum
R. multithec-atum
R. sociale
Cordylodus angulatus
Cordylodus Indstromi
Acerocare
C. intrmedius
C. proavus
Cordylodus andresi
Peltura
"Procono-dontus"
Leptoplastus
Parabolina spinulosa
Homagnostus/ Olenus
Agnostus pisiformis
Westergaardodina
Paradoxides forchammeri
Paradoxides paradoxissimus
Eccaparadoxides oelandicus
Naziya Fm.
Koporiye Formation
Tosna Formation
Lomashka Formation
Ladoga Formation Upper Mb.
Ladoga Fm.
Sablinka Formation
Rebrovo Beds
Gertovo Beds
Lower Unit
Varangu Fm.
Türisalu Formation (incl. Tabasalu, and Toolse members)
Orasoja Mb.
Vihula Member
Rannu Mb.
Upper Maardu Member
Suurjõgi Member
Lower Maardu Member
Tsitre Formation
Ülgase Formation
Salantai Member
Ladushkino Fm.
?
Veselovsk Formation
Deimena Fm.
Kybartai Fm.
Bugino Formation
Pestovo Fm.
Nikolskoye Formation
Tolbukhino Formation
Urdom Formation

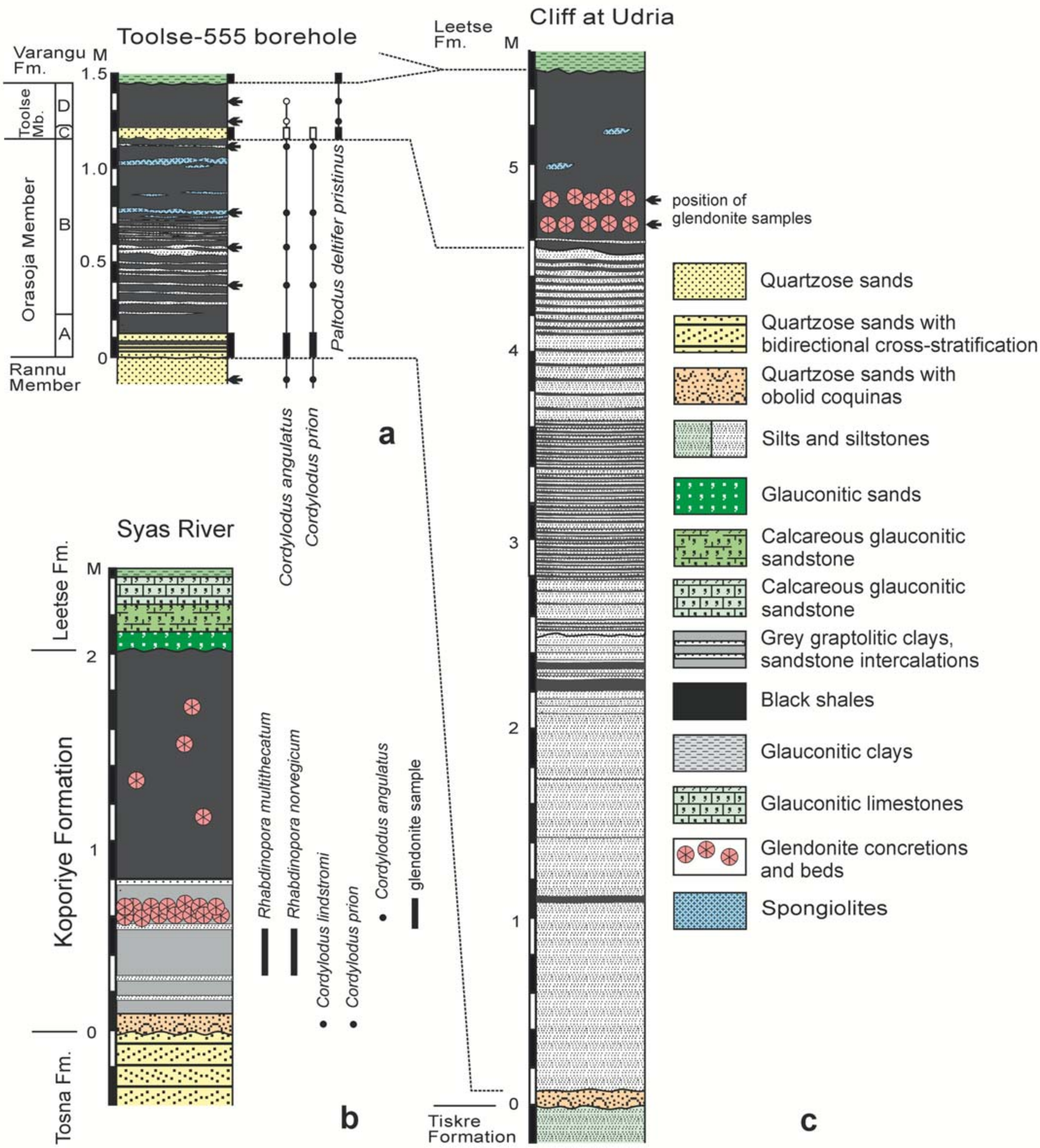
Toolse-555 borehole
Varangu Fm.
M
1.5
1.0
0.5
0
Toolse Mb.
D
C
B
A
Orasoja Member
Rannu Member
Cordylodus angulatus
Cordylodus prion
Paltodus deltifer pristinus
a
Cliff at Udria
Leetse Fm.
M
5
4
3
2
1
0
position of glendonite samples
Tiskre Formation
c
Syas River
Leetse Fm.
M
2
1
0
Koporiye Formation
Tosna Fm.
Rhabdinopora multithecatum
Rhabdinopora norvegicum
Cordylodus lindstromi
Cordylodus prion
Cordylodus angulatus
glendonite sample
b
Quartzose sands
Quartzose sands with bidirectional cross-stratification
Quartzose sands with obolid coquinas
Silts and siltstones
Glauconitic sands
Calcareous glauconitic sandstone
Calcareous glauconitic sandstone
Grey graptolitic clays, sandstone intercalations
Black shales
Glauconitic clays
Glauconitic limestones
Glendonite concretions and beds
Spongiolites

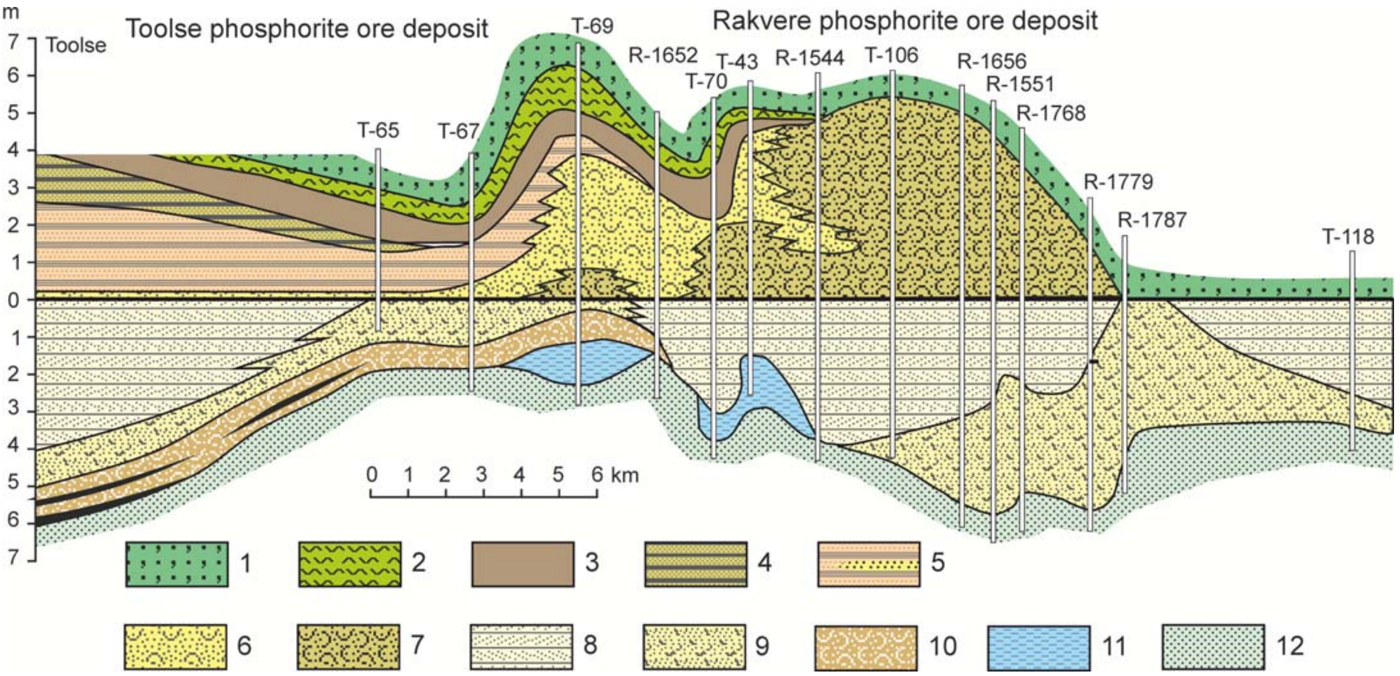

m
Toolse
Toolse phosphorite ore deposit
Rakvere phosphorite ore deposit
T-65
T-67
T-69
R-1652
T-70
T-43
R-1544
T-106
R-1656
R-1551
R-1768
R-1779
R-1787
T-118
0 1 2 3 4 5 6 km
1
2
3
4
5
6
7
8
9
10
11
12

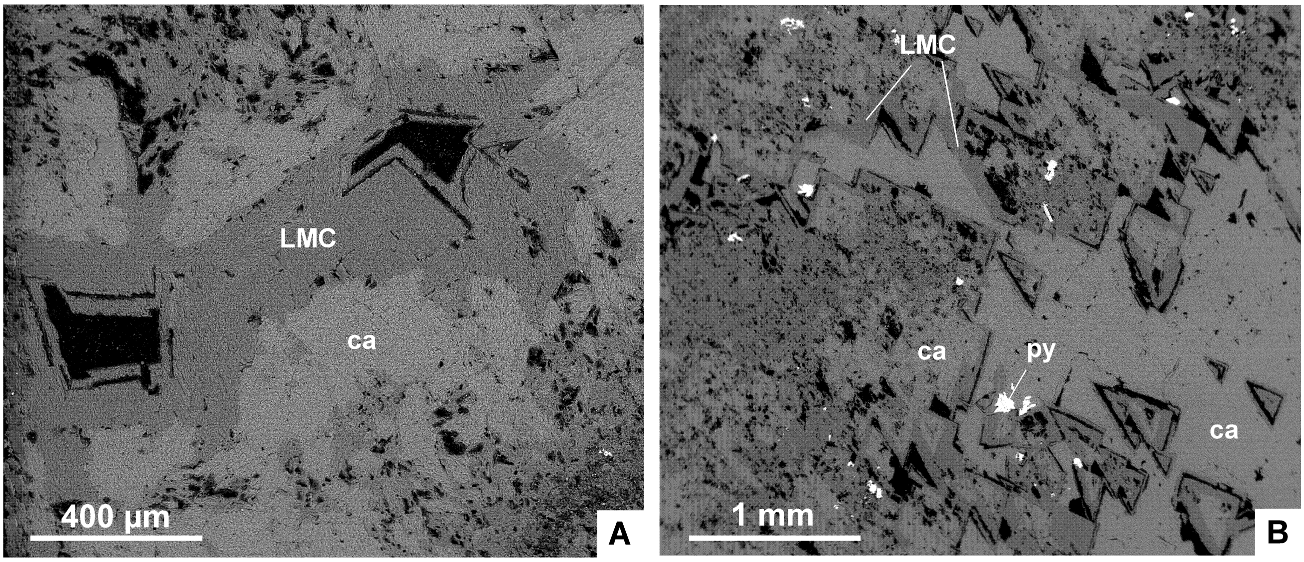
LMC
ca
400 µm
A
LMC
ca
py
ca
1 mm
B

	MgO	CaO	Fe_2O_3	Na_2O	K_2O
SP1a	23,4	42,9	11,6	0,04	1,3
SP1b	23,6	42,8	11,4	0,06	1,3
SP1c	23,5	43	12,1	0,05	1,2
SP1d	24,2	42,8	11,9	0,05	1,2
SP2a	0,9	92,6	1,1	0,01	0,3
SP2b	0,7	83,5	0,8	0,01	0,3
SP2c	0,8	88,6	1,2	0,01	0,4
DMA	0,8	95,3	1,3	0,01	0,4

MnO	Sr (ppm)	
0,6	52	low-Mg calcite
0,7	52	low-Mg calcite
0,7	50	low-Mg calcite
0,7	56	low-Mg calcite
0,4	120	calcite
0,5	116	calcite
0,4	132	calcite
0,2	124	calcite

sample	δ^{13}Ccarb V-PDB	δ^{18}Ocarb V-PDB
DMA-1	-1,23	-6,68
DMA-2	-1,18	-6,82
DMA-3	-1,34	-6,77
DMA-4	-3,82	-8,21
DMA-5	-1,45	-7,11
SP-1	0,33	-6,53
SP-2	-2,59	-5,71
SP-3	-4,9	-8,06
SP-4	0,6	-6,21
SP-5	0,35	-6,81

sample	East Baltic Stage	Conodont Zone	δ18O (‰VSMOW)	1 std. Dev.
OL-A2	Pakerort Stage	*Cordylodus angulatus*	14,42	0,14
OL-B 3-4	Pakerort Stage	*Cordylodus angulatus*	14,44	0,26
OL-C	Pakerort Stage	*Paltodus deltifer* (*P. d. pristinus* Subzone)	14,66	0,29
